# Acute kidney injury in chronic liver disease: Causes and prevalence in a cross-sectional study

**DOI:** 10.6026/973206300213118

**Published:** 2025-09-30

**Authors:** Pankaj Kumar Gupta, Rahul Soni, Yasha Bandil, Madhu Saurabh Singh Dhurvey, Sachin Parmar

**Affiliations:** 1 Deprtment of General Medicine, Birsa Munda Government Medical College, Shahdol, Madhya Pradesh, India; 2 Department of Pulmonary Medicine, Birsa Munda Government Medical College, Shahdol, Madhya Pradesh, India; 3 Department of Ophthalmology, Birsa Munda Government Medical College, Shahdol, Madhya Pradesh, India; 4 Department of Community Medicine, V.K.S. Government Medical College, Neemuch, Madhya Pradesh, India

**Keywords:** Chronic liver disease, acute kidney injury, hepatic decompensation, sepsis, alcoholic liver disease

## Abstract

Chronic liver disease (CLD) is a prevalent condition with significant systemic complications and acute kidney injury (AKI) is a common and serious complication that worsens prognosis. This prospective observational study, conducted from November 2019 to June 2021 at G.R. Medical College, Gwalior, included 100 CLD patients. The study found that 30% of patients developed AKI, with sepsis as the most common precipitating factor. Severe renal impairment (Stage 3 AKI) was present in 46.6% of AKI cases. Alcohol was the leading etiology of CLD and the timely diagnosis and management of AKI in CLD patients are essential to improving outcomes and reducing mortality.

## Background:

Chronic liver disease (CLD) is a prevalent medical condition impacting humanity. Its correlation with diseases affecting other vital organs, such as the kidneys, renders it a more catastrophic condition. Acute kidney injury (AKI) significantly impacts the prognosis and outcomes of patients with chronic liver disease (CLD); therefore, it is crucial to prevent the onset of AKI and promptly identify the precipitating factors to facilitate early interventions [1]. Chronic liver disease is often complicated by acute kidney injury, exacerbating the prognosis [2]. The two predominant causes of acute kidney injury (AKI) in patients with chronic liver disease are hepatorenal syndrome type AKI (HRS-AKI), a spectrum of disorders associated with prerenal chronic liver disease and acute tubular necrosis (ATN). Distinguishing between these two conditions is crucial due to the variance in treatment approaches. Prerenal acute kidney injury (AKI) is a relatively benign condition characterized by a favorable response to plasma volume expansion, whereas acute tubular necrosis (ATN) necessitates more targeted management and is linked to considerable mortality [3]. While prerenal azotemia resulting from severe hypoalbuminemia appears to be the initial phase of acute kidney injury (AKI), distinguishing it from hepatorenal syndrome is essential. The differentiation between hepatorenal syndrome (HRS) and acute tubular necrosis (ATN) is challenging [4]. Consequently, the manifestation of renal dysfunction in patients with chronic liver disease necessitates not only prompt identification but also accurate diagnosis and optimal management [5]. Physiological alterations in patients with chronic liver disease elevate their risk of developing acute kidney injury. Systemic and splanchnic vasodilation typically leads to a subsequent decrease in effective circulating volume, which may critically impair renal perfusion in patients with cirrhosis and portal hypertension [6]. Etiologies of acute kidney injury in cirrhosis include [7]: (i) Prerenal acute kidney injury (AKI), characterized by hypovolemia resulting from gastrointestinal hemorrhage, excessive diuretic therapy, infections, or lactulose-induced diarrhea; (ii) Hepatorenal syndrome-type AKI (HRS-AKI), defined as a potentially reversible deterioration of renal function unresponsive to volume resuscitation, resulting from renal vasoconstriction in the absence of identifiable alternative causes; (iii) Intrinsic causes (e.g., Acute Tubular Necrosis); (iv) Post-renal etiologies. Chronic liver disease results in an immuno-compromised condition, thereby elevating the risk of infections in these patients [8]. Consequently, prompt assessment and intervention for infection is essential for patients with cirrhosis who experience acute kidney injury (AKI) [9]. Therefore, it is of interest to report acute kidney injury in chronic liver disease.

## Materials and Methods:

Present study is a prospective observational study conducted in the Department of General Medicine, G.R. Medical College, Gwalior (M.P.), on an inpatient (IPD) basis. Subjects are selected from J.A. Group of Hospitals, Gwalior.

## Sample size:

100

## Type of study:

Prospective and observational studies.

## Duration of study:

Nov. 2019 to June 2021

## Methodology:

Patients admitted with clinical features of chronic liver disease or Cirrhosis will be evaluated with S.bilirubin ,AST, ALT, Total protein, Albumin, ALP, Platelet count, S.creatinine, S.Urea, Prothrombin time and INR value. Detailed history and physical examination will be performed and Data will be collected using a standardized proforma. USG abdomen will be done to evaluate liver morphology and kidney size Hep B and C status will be assessed. Investigations will be collected and documented to make a definite diagnosis of acute kidney injury and chronic liver disease. Document the probable causes and outcome of patients who develops AKI in the background of CLD.

## Inclusion criteria:

[1] Age more than 18 years.

[2] Diagnosed case of chronic liver disease.

[3] Patient of acute kidney injury in chronic liver disease.

## Exclusion criteria:

[1] Known case of CKD.

[2] Patients of chronic diseases such as active tuberculosis, malignancy, coronary artery disease (CAD) , multiple organ dysfunction syndrome (Mods) , Acute dengue fever, valvular heart disease.

## Results:

In this study, among 100 patients with chronic liver disease (CLD), the demographic and clinical profile is presented in Table 1 (see PDF). The majority were males, comprising 70% of the study population, while females accounted for the remaining 30%. Age-wise distribution showed that most patients (63%) were between 46-60 years, followed by 22% in the 30-45 age groups. Only 8% of patients were younger than 30 years and 7% were older than 60. Based on the Child-Pugh classification, 60% of patients were in Class C, indicating advanced liver dysfunction. Class B was seen in 39% of patients and only 1% fell under Class A. Alcohol was the predominant cause of CLD, accounting for 59% of cases, followed by hepatitis B virus (20%), hepatitis C virus (6%) and other less common causes such as NASH-related liver disease (2%), portal vein thrombosis (5%), unknown causes (7%) and Wilson's disease (1%). Table 2 (see PDF) shows Cause of CLD in Malesamong the male patients with chronic liver disease, alcohol emerged as the leading etiology, responsible for 84.3% of the cases. Other causes included hepatitis B infection (8.6%), portal vein thrombosis (2.8%) and NASH-related liver disease (2.8%). Wilson's disease was a rare etiology, observed in 1.4% of male cases. These findings underscore the significant contribution of alcohol to the burden of CLD among male patients. Table 3 (see PDF) show Hepatic Decompensation and Renal Dysfunction in CLD Patients (n = 100). Signs of hepatic decompensation were common among CLD patients, with ascites present in 78% of cases, followed by pedal edema (67%), jaundice (52%) and gastrointestinal bleeding (22%). Other clinical signs included altered sensorium (20%), dilated abdominal veins (18%), alopecia (10%), palmar erythema (8%) and testicular atrophy (4%). Acute kidney injury (AKI) was observed in 30% of the patients. The most common causes of renal dysfunction were sepsis (40%), gastrointestinal bleeding (26.7%), use of diuretics (13.3%), diarrhea (10%), paracentesis (6.6%) and vomiting (3.3%). Among those with AKI, 26.6% were classified as Stage 1, another 26.6% as Stage 2 and a majority (46.6%) presented with Stage 3 AKI, indicating severe renal impairment in a significant subset of CLD patients.

## Discussion:

This study is conducted in the Department of General Medicine at Gajra Raja Medical College and J.A. Group of Hospitals in Gwalior, Madhya Pradesh, to examine the clinical profile of acute kidney injury in patients with chronic liver disease. Our study includes 100 patients with chronic liver disease, selected according to established inclusion and exclusion criteria and the findings are compared with analogous studies. The current study comprises a total of 100 patients, including 70 males and 30 females. The patients' ages range from 18 to 85 years. The male to female ratio is 7:3 and the average age is 48.1 years. A comparable study conducted by Purohit *et al.* [2] at Shri Aurobindo Institute of Medical Sciences in Indore revealed that among 100 patients, 87 were male and 13 were female, resulting in a male to female ratio of 6.6:1. A comparable study conducted by Das *et al.* [5] at Calcutta National Medical College & Hospital, Kolkata, revealed that the majority of patients were in their forties, with a mean age of 43.58 years and a predominance of male patients. In several other studies, males were more frequently involved than females, such as in the research by Andrew *et al.* [6] where the median (first quartile, third quartile) age was reported. The mean age of the entire cohort was 58 years, with a range of 50 to 65 years. The predominant participants were male (71%), of Caucasian descent (93%) and non-Hispanic (87%). The majority of patients presented to the hospital at an advanced stage. In our study, 60% of the 100 cases were classified as Child Pugh Class C, while 39% were classified as Class B. Only 1% of cases belong to Child Pugh Class A. This outcome parallels that of Purohit *et al.* [2] where the majority of cases were classified as Child Class C (56%), followed by Class B (34%) and Class A 10%). In this study of 30 cases of chronic liver disease (CLD) that developed acute kidney injury (AKI), the predominant cause of AKI was identify as sepsis (40%), followed by gastrointestinal bleeding (26.7%) and inappropriate diuretic use (13.3%). Paracentesis and diarrhea accounted for 6.6% and 10% of cases, respectively, whereas severe vomiting was the etiology of AKI in 3.3% of cases. The current study identified chronic alcoholism as the predominant cause of chronic liver disease in males, accounting for 84.3%, followed by hepatitis B virus infection at 8.6%. Portal vein thrombosis and NASH-related hepatitis accounted for 2.8% of chronic liver disease cases in patients each, whereas Wilson's disease was responsible for 1.4% of cases. The current study identified hepatitis B virus infection as the predominant cause of chronic liver disease in females, accounting for 46.6%, followed by Hepatitis C virus infection (20%). Portal vein thrombosis accounted for 10% of chronic liver disease cases, whereas the etiology of chronic liver disease remained unidentified in 23.3% of cases. The findings were analogous to those of Purohit *et al.* [2] where alcoholic cirrhosis constituted the predominant etiology at 77%, followed by hepatitis B at 10%, hepatitis C at 3%, NASH at 2% and Wilson's disease at 1%. Jaiprakash *et al.* [7] identified that the predominant etiology of cirrhosis was chronic alcoholism (29.8%), succeeded by cryptogenic causes (25.3%) and chronic hepatitis B (24.1%). Other infrequent causes comprised chronic hepatitis C (7.3%), primary biliary cirrhosis, non-alcoholic fatty liver disease, auto-immune hepatitis, the combined effects of alcoholism and hepatitis B and Wilson's disease, listed in descending order of prevalence. Das *et al.* [8] found that the predominant etiology of cirrhosis was alcohol, reveals, among study population, 34 (68%) patients suffered from Alcoholic liver disease, 7 (14%) patients had chronic Hepatitis-B, 3 (6%) patients had chronic Hepatitis-C, 3 (6%) patients had Non-alcoholic fatty liver disease, 2 (4%) patients had Wilson's Disease and 1 (2%) patients had autoimmune hepatitis. Additional research conducted on patients with cirrhosis revealed that alcohol was the predominant etiology of the condition. In the study by Sharma *et al.* [9] alcohol was identified as the etiology of chronic liver disease (CLD) in 62.9% of cases, followed by hepatitis B virus (HBV) infection at 10.1% and non-alcoholic steatohepatitis (NASH) in less than 10% of cases. A study conducted by Shah *et al.* [10] found that alcohol was present in 48%, NASH in 26%, HBV in 10%, HCV in 6% and 7% had other causes of chronic liver disease (CLD). Indicators of hepatic decompensation: In this study, ascites was the predominant indicator of hepatic decompensation (78%), followed by pedal edema (67%). Jaundice was observed in 52% of patients. Altered sensorium and gastrointestinal bleeding were observed in 20% and 22% of patients, respectively.

18% of patients exhibited dilated abdominal veins, whereas 10% displayed alopecia. Palmar erythema was observed in merely 8% of patients, whereas 4% exhibited testicular atrophy. Purohit *et al.* [2] found that the predominant indicator of hepatic decompensation was jaundice at 78%, followed by ascites at 70%, hepatic encephalopathy at 37% and upper gastrointestinal bleeding at 30%. In a comparable study conducted by Banait *et al.* [11] it was determined that the predominant sign of decompensation was pedal edema (93.5%), succeeded by gastrointestinal bleeding (63.5%). Additional manifestations included icterus (62%), parotid swelling (43.5%), dilated veins (37%), alopecia (29.5%), flapping tremor (28.5%), altered sensorium (26%), palmar erythema (17.5%), spider nevi (13.5%) and testicular atrophy (13%).Prevalence of AKI: In this study, 30% of 100 patients with chronic liver disease (CLD) developed acute kidney injury (AKI). A comparable study conducted by Gines *et al.* [12] in the Centre for Hepatology, Royal Free and University College Medical School, London, The prevalence of acute kidney injury (AKI) among hospitalized patients with chronic liver disease is approximately 20%.Prakash*et al.* [7] found that the incidence of acute kidney injury (AKI) in chronic liver disease is 24.5%.The study identified the causes of renal dysfunction in chronic liver disease (CLD) among 30 cases that developed acute kidney injury (AKI). The predominant cause was sepsis (40%), followed by gastrointestinal bleeding (26.7%) and inappropriate diuretic use (13.3%). Paracentesis and diarrhea accounted for 6.6% and 10% of cases, respectively, whereas severe vomiting was the etiology of AKI in 3.3% of cases. The findings closely resemble those of Purohit *et al.* [2] where the predominant precipitating factor was sepsis at 33%, followed by spontaneous bacterial peritonitis (SBP) at 18%, upper gastrointestinal (UGI) bleeding at 10%, diuretics at 9%, urinary tract infections (UTI) at 8% and paracentesis, diarrhea, vomiting, bacteremia and pneumonia at 4% each, with aminoglycosides at 1%.Numerous studies indicate that sepsis is one of the most prevalent causes of acute kidney injury (AKI) in patients with cirrhosis, while other causes rank differently. Jaiprakash *et al.* [7] similarly observed that sepsis was the predominant cause of acute kidney injury (AKI) in their study. Watt *et al.* [13] similarly observed that bacterial infection was the predominant precipitating factor (48%) for the onset of AKI in cirrhosis, followed by gastrointestinal bleeding (33%). In this study, among 30 cases of chronic liver disease (CLD) that developed acute kidney injury (AKI), 46.6% exhibited stage 3 AKI, while 26.6% presented with stage 1 and stage 2 AKI, respectively. This aligns with the findings of the study by Purohit *et al.* [2] which identified the highest percentage of patients in AKI stage III at 40%, followed by stage I at 36% and stage II at 24%.In a comparable study conducted by Belcher *et al.* [14], 49% of cirrhosis patients exhibited AKIN stage 3 acute kidney injury, while the prevalence of stage 1 and stage 2 acute kidney injury was 26% and 24%, respectively Rognant [15]. The occurrence of AKI in patients with severe cirrhosisis a common event associated with a worsening of the prognosis. In our study, the in-hospital mortality rate for patients with chronic liver disease (CLD) complicated by acute kidney injury (AKI) was significantly elevated at 56.6%, compared to 8.5% for those without AKI, with a p-value of less than 0.05.This aligns with the findings of the study by Jenq *et al.* [16] which reported that among 134 patients with cirrhosis admitted to the ICU, the mortality rates were 32.1% for those without AKI, 68.8% for RIFLE-R, 71.4% for RIFLE-I and 94.8% for RIFLE-F. In a comparable study conducted by Belcher *et al.* [15] the overall mortality rate in cirrhosis complicated by acute kidney injury (AKI) was determined to be 26%. A notable stage-dependent response was observed, with mortality rates for peak AKIN stages 1, 2 and 3 at 2%, 15% and 44%, respectively.

## Conclusion:

Chronic liver disease predominantly affects middle-aged males with alcohol as the leading etiology, with most patients presenting at advanced stages (Child-Pugh Class C) and exhibiting hepatic decompensation including ascites and pedal edema. Acute kidney injury occurred in 30% of CLD cases, primarily triggered by sepsis, with a significant proportion classified as Stage 3 severity and associated with markedly higher in-hospital mortality rates. Early diagnosis, close monitoring, and timely management of renal complications in CLD patients is essential to reduce associated morbidity and mortality.
